# Cloudy Dialysate as the Initial Presentation for Lymphoma

**DOI:** 10.1155/2018/2192043

**Published:** 2018-10-09

**Authors:** Sriram Sriperumbuduri, Deborah Zimmerman

**Affiliations:** ^1^Division of Nephrology, Department of Medicine, Ottawa Hospital, Ottawa, ON, Canada; ^2^University of Ottawa, Kidney Research Centre of the Ottawa Hospital Research Institute, Ottawa, ON, Canada

## Abstract

Turbid dialysate in a patient on peritoneal dialysis is usually due to peritonitis and almost all these patients are started on empirical antibiotics pending cultures. However, in few of them with culture negative fluid, this could represent other etiologies like chyle, which requires more intensive investigations, and analysis of fluid itself reveals some rare diagnosis. We present one such report of chylous ascites with prompt investigation leading to a diagnosis of malignancy in a peritoneal dialysis patient.

## 1. Introduction

The most common cause of cloudy dialysate in patients on peritoneal dialysis (PD) is bacterial peritonitis such that the home dialysis unit policies and procedures include protocols for specimen collection and initial antibiotic therapy algorithms to avoid delays in diagnosis and treatment. However, turbid dialysate may be secondary to other etiologies including increased neutrophils due to intra- or juxtaperitoneal inflammation, red blood cells, malignant cells, and an elevated triglyceride content. Chylous ascites is a rare cause of turbid dialysate and has been associated with lymphoma, pancreatitis, and superior vena cava syndrome.

## 2. Case Report

86-year-old man with a history of hypertension and type 2 diabetes mellitus had been treated for end stage kidney disease with continuous cycling peritoneal dialysis since February 2017. He presented to the home dialysis unit complaining of difficulties with initial drain alarms on his cycler for the last 2 nights and “whitish” dialysate. He denied abdominal pain or constitutional symptoms aside from weight loss associated with resolution of peripheral edema. He did not have any previous episodes of peritonitis or history of TB contact. His examination was unremarkable including normal vital signs and lack of abdominal tenderness.

As per out unit peritonitis protocol, 1L of 2.5% Dianeal was allowed to dwell for 2 hours and the effluent was sent for analysis including cell count, differential, bacterial, and mycobacterial cultures. Given the “milky” appearance of the fluid, triglycerides were also ordered. He received empiric intraperitoneal antibiotics including ceftazidime and vancomycin. Total nucleated cell count was 354 *∗* 10^6^/L with 87% lymphocytes, 8% monocytes, and 3% neutrophils. Cultures were negative. Triglyceride (TG) concentration was 6.3 mmol/L (557 mg/dl). Based on the elevated TG concentration he underwent a CT scan with contrast of the abdomen and a second dialysate sample was sent for cell count, TG, cytology, and flow cytometry (the dialysate was no longer cloudy). He was found to have a mildly enlarged spleen and multiple enlarged lymph nodes in the mesentery, retroperitoneum, and inguinal regions including a cluster of enlarged nodes forming a conglomerate retroperitoneal mass suggestive of lymphoma. There was a moderate increase in density of the mesentery, possibly on the basis of lymphatic obstruction. His total nucleated cell count remained elevated at 420 with 96% lymphocytes; TG concentration was only 0.21 mmol/L. Cytology was negative for malignant cells. Flow cytometry of the dialysate showed predominately monotypic B cells with lambda light chain restriction that coexpressed CD20 and CD19 but lacked CD5 and CD10 suggestive of a monoclonal lymphoid process. An inguinal lymph node biopsy revealed predominant diffuse to nodular pattern of small monotonous lymphocytes, suggestive of B cell lymphoma. Immunohistochemical stain was positive for CD20 (diffuse), BCL2 (diffuse), and few remaining CD21 (FDC). It was negative for CD3, CD5, CD10, CD23, and C43. Kappa and lambda stains were nonspecific. Ki67 proliferation index was less than 5%. Final diagnosis was monoclonal B cell lymphoproliferative disorder, likely of low grade. He is being followed by the malignant hematology team, with no active treatment. From a PD perspective, the patient had no further episodes of chylous ascites and remains on CCPD.

## 3. Discussion

Chylous ascites is rare but is associated with several potentially life-threatening diagnoses including tuberculosis, pancreatitis [1], and malignancy [2]. The use of calcium channel blockers (CCBs), surgical trauma, and superior vena cava obstruction [3, 4] has also presented with chylous ascites in peritoneal dialysis patients. In PD patients, the cloudy dialysate may lead to initiation of unnecessary antibiotics and delayed investigations. Negative cultures and a differential count that is predominately lymphocytes should prompt further investigation. In our patient's case, the atypical appearance of the cloudy dialysate led immediately to further investigation but he continued to receive antibiotics.

Case series of lymphoma presenting with chylous ascites have been published in patients without ESKD [5, 6]. Earlier reports suggested a poor prognosis with this complication in patients with lymphoma [7], but more recent studies show improved outcomes with the advent of newer chemotherapy regimens [8]. Very few case reports exist about lymphoma leading to chylous ascites in PD patients [9, 10]. Daily lavage of peritoneum actually facilitates diagnosis of this condition at a potentially earlier stage in these patients, as in our case where the high triglyceride level was detected at the first appearance of cloudy effluent. After a few days, the cloudiness disappeared with a decrease in triglyceride levels. This may be due to intermittent lymphatic obstruction leading to leak of chyle into peritoneal cavity as the patient's diet was never modified. Flow cytometry facilitated the diagnosis of a monoclonal lymphoid process confirmed by lymph node biopsy suggestive of low grade non-Hodgkin lymphoma.

Management options in chylous ascites include discontinuing offending medications (CCBs), altering the diet to predominantly medium chain triglycerides (MCT), somatostatin analogues (they increase splanchnic arteriolar resistance and decrease lymph flow) [11], and instituting total parenteral nutrition (TPN). The median time to resolution of chylous ascites was 28 days with a low-fat diet supplemented with MCT and 10 days with TPN [12].

In conclusion, a single episode of cloudy dialysate with a lipid consistency in PD patients should prompt further evaluation.
